# Adverse Early-Life Factors Associated with Clonal Hematopoiesis of Indeterminate Potential in Later Life

**DOI:** 10.3390/biomedicines14061366

**Published:** 2026-06-17

**Authors:** Yuefeng Yu, Junxue Wang, Ying Sun, Bowei Yu, Xiao Tan, Yingli Lu, Fangzhen Xia, Ningjian Wang

**Affiliations:** 1 Department of Endocrinology and Metabolism, Pudong Gongli Hospital, Shanghai University of Medicine & Health Sciences, Shanghai 201318, China; yuyuefeng981125867@163.com; 2 Institute and Department of Endocrinology and Metabolism, Shanghai Ninth People’s Hospital, Shanghai JiaoTong University School of Medicine, Shanghai 200011, China; jxwang0104@163.com (J.W.); sunying_0216@126.com (Y.S.); ybw475773130@163.com (B.Y.); luyingli@sjtu.edu.cn (Y.L.); 3 School of Public Health, Zhejiang University, Hangzhou 310027, China; xiao.tan@neuro.uu.se; 4 Department of Medical Sciences, Uppsala University, 753 10 Uppsala, Sweden

**Keywords:** clonal hematopoiesis of indeterminate potential, early-life factors, ASXL1, sexual abuse, recurrent antibiotic use

## Abstract

**Background:** Clonal hematopoiesis of indeterminate potential (CHIP) can lead to adverse outcomes and may begin early in life. This study aimed to investigate the association between early-life events and CHIP. **Methods:** In total, 456,658 participants from U.K. Biobank without baseline hematologic malignancies were enrolled. Exposures included 17 early-life events, including reproductive, childhood adversity, and pre-adulthood development factors. CHIP was derived from whole-exome sequencing for mutations in 74 driver genes. Logistic regressions were used to estimate associations between early-life events and the presence of any CHIP or gene-specific CHIP mutations. **Results:** Overall, 17,513 (3.8%) individuals with any CHIP were identified, among which the most common subtype was *DNMT3A* (2.4%), followed by *TET2* (0.6%) and *ASXL1* (0.4%). Compared with participants without sexual abuse in childhood, those who experienced such abuse were positively associated with CHIP (OR 1.35, 95% CI 1.02–1.80), especially among *ASXL1*, *JAK2*, and *TP53* mutations. Long-term/recurrent antibiotic use as a child or teenager was positively associated with CHIP (OR 1.11, 95% CI 1.02–1.21), especially among *DNMT3A*, *ASXL1*, and *EP300* mutations. Sex-specific differences were observed, including sexual abuse associated with *ASXL1*-CHIP in males and *JAK2/TP53*-CHIP in females and long-term/recurrent antibiotic use associated with *DNMT3A/EP300*-CHIP in males and *ASXL1*-CHIP in females. Furthermore, we identified circulating proteomic biomarkers shared by six pairs of early-life factors and gene-specific CHIP mutations, including B2M for sexual abuse and *JAK2*-CHIP. **Conclusions:** Early-life factors, especially sexual abuse and long-term/recurrent antibiotic use, were positively associated with the presence of CHIP, particularly among specific gene mutations, offering potential targets for susceptibility and pathogenesis exploration.

## 1. Introduction

Clonal hematopoiesis of indeterminate potential (CHIP) refers to the clonal expansion of blood cells driven by somatic mutations within the hematopoietic stem cells in individuals without diagnosed hematologic neoplasia, dysplasia, or cytopenia [1,2]. According to the latest World Health Organization (WHO) and International Consensus Classification (ICC) guidelines, CHIP somatic mutations most frequently occur in *DNMT3A*, *TET2*, *ASXL1*, or *JAK2*, the epigenetic and transcriptional regulation genes, and in *SF3B1*, *SRSF2*, or *U2AF1*, the mRNA splicing genes, as well as in *TP53* or *PPM1D*, the DNA damage repair genes [3,4]. Due to the widespread availability and decreased cost of whole-exome sequencing (WES), identification of CHIP-associated mutations can be carried out in large cohorts [5]. Notably, these mutations are ubiquitous even though individuals are apparently healthy and have normal or only mildly perturbed blood counts [6]. It has been confirmed that individuals with CHIP are at an increased risk of hematologic cancer; solid malignancy; cardiovascular, pulmonary, and metabolic diseases, as well as all-cause mortality [7,8,9]. As the range of adverse health outcomes secondary to CHIP increases, it is crucial to identify risk factors for the occurrence of CHIP.

Recent studies have elucidated the potential effects of environmental factors on CHIP. For instance, analyses from the U.K. Biobank have indicated that current smoking status is most related to *ASXL1* mutation [10]. Previous exposure to cancer therapies was significantly associated with CHIP mutations in *PPM1D* and *TP53* [11]. First responders who were exposed to aerosolized dust, gases, and potential carcinogens were demonstrated to be at a higher risk of CHIP, especially in *DNMT3A* and *TET2* [12].

However, to date, exploration of early-life exposures and CHIP occurrence is still lacking. Notably, as CHIP is an age-dependent disorder, its occurrence can span decades [13], and the etiologically relevant window for CHIP may begin early in life. Individuals who experience early adverse events tend to have unfavorable lifestyles (i.e., smoking or risky human immunodeficiency virus behaviors), health-related diseases (i.e., diabetes), and elevated systemic inflammation levels [14,15], all of which are well-characterized risk factors for CHIP [4,10,16,17].

Therefore, to address these gaps, we focused on early-life events, including reproductive, childhood adversity, and pre-adulthood development factors, to investigate their association with CHIP occurrence. Furthermore, to explore potential mechanisms, we examined associations among early-life events, circulating proteomic biomarkers, and gene-specific CHIP.

## 2. Methods

Early-life events, circulating proteomics, and covariates including demographic characteristics, lifestyle behaviors, and medical information were obtained through the U.K. Biobank. CHIP data were obtained from a recent study [18]. We conducted the subsequent analyses.

### 2.1. Study Cohort

The U.K. Biobank is a prospective cohort study consisting of over 500,000 adults aged 40–69 years across England, Scotland, and Wales, recruited from 22 assessment centers between 2006 and 2010 [19]. The UKB received ethical approval from the U.K. National Health Service, the National Research Ethics Service—North West, the National Information Governance Board for Health and Social Care in England and Wales, and the Community Health Index Advisory Group in Scotland. This study was approved by the U.K. Biobank (application number 77740 and 1262160).

This study collected data from 502,417 individuals in the U.K. Biobank. We excluded 45,203 participants lacking exome sequencing data for CHIP or with hematologic malignancies at baseline. Finally, after excluding participants with missing data for early-life events, a total of 456,658 individuals were included in the main analyses (Figure S1).

### 2.2. Early-Life Events

At baseline, participants provided information, such as demographic characteristics, lifestyle behaviors and medical information, by completing a comprehensive and standardized touchscreen questionnaire and a verbal interview. Events occurring during infancy and childhood were included in the category of early-life factors. Some issues related to sexual development were included in the category of gender-specific factors. Additionally, nearly one-third of the participants took part in the online follow-up in 2016 and responded to questions about traumatic events, including childhood adverse events.

In this study, we included 17 early-life events covering three aspects. The first aspect consisted of five reproductive factors, including birth weight (continuous), born by caesarian section, part of a multiple birth, breastfed as a baby, and maternal smoking around birth. The second aspect consisted of five childhood adversities, including emotional neglect (rarely/never felt loved as a child), sexual abuse (often/very often sexually molested as a child), physical abuse (often/very often physically abused by family as a child), emotional abuse (often/very often felt hated by a family member), and physical neglect (rarely/never taken to doctor when needed as a child). The third part consisted of seven pre-adulthood development factors, including adopted as a child, comparatively thinner/plumper at age 10, comparatively shorter/taller at age 10, relatively younger/older when growing first facial hair, relatively younger/older when voice broke, age when periods started (menarche) (continuous), and long-term/recurrent antibiotic use as a child or teenager. Long-term/recurrent antibiotic use as a child or teenager was defined as receiving three or more courses of antibiotics per year during childhood or adolescence. (Table S1).

### 2.3. Clonal Hematopoiesis of Indeterminate Potential

CHIP identification was derived from whole-exome sequencing (WES) of peripheral blood samples, performed on the Illumina NovaSeq 6000 platform at the Regeneron Genetics Center (Tarrytown, NY, USA) [18,20]. CHIP mutations within 74 driver genes were called, which was widely applied in CHIP studies in UKB cohort (Table S2). Putative somatic variants were identified from aligned sequencing (CRAM) files by Mutect2 [21,22]. Subsequently, quality control filtered out false-positive CHIP mutations as much as possible: (1) retaining mutations that simultaneously fulfilled total allele depth (DP) ≥ 20, minimum allele depth (minAD) ≥ 5, and bidirectional reads (F1R2 and F2R1 ≥ 1); (2) choosing bona fide biallelic variants from multiallelic variants; (3) excluding variants with <20 individuals or those not associated with either age or the TERT promoter (rs7705526); (4) excluding germline variants that failed the binomial test; and (5) excluding genes not positively associated with age, which were not observed in myeloid cancer cases.

Any CHIP, defined as a variant allele fraction (VAF) ≥ 2%, and large CHIP, defined as VAF ≥ 10%, were considered as the primary outcomes in this study. Furthermore, we conducted separate analyses for 30 gene-specific CHIP subtypes that were present in >30 individuals, including *DNMT3A*, *TET2*, *ASXL1*, *PPM1D*, *TP53*, *SRSF2*, *NF1*, *JAK2*, *SF3B1*, and *CREBBP*.

### 2.4. Circulating Proteomic Measurements

Plasma proteomic data were obtained from the U.K. Biobank Pharma Proteomics Project (UKB-PPP), which profiled plasma samples using the antibody-based Olink Explore 3072 proximity extension assay. In this study, 45,507 randomly selected baseline participants with available plasma proteomic data were included. We used the UKB-PPP quality-controlled normalized protein expression (NPX) data. The UKB-PPP quality-control procedure included removal of control samples, withdrawn or unprocessed samples, outlier samples, measurements with quality-control or assay warnings, and likely sample swaps. After quality control, 2923 proteins remained across eight panels, including cardiometabolic, inflammation, neurology, and oncology panels and their corresponding expansion panels. Proteins with more than 50% missing values were further excluded, including GLIPR1, NPM1, and PCOLCE, leaving 2920 proteins for analysis. NPX values were inverse-rank normalized before analysis.

### 2.5. Statistical Analysis

Baseline characteristics were presented as medians (interquartile range) for continuous variables and as percentages for categorical variables. Comparisons of baseline characteristics across the categories of CHIP were made using Student’s t-test for continuous variables or the Pearson chi-squared test for categorical variables.

Logistic regression models were employed to calculate odds ratios (ORs) and 95% confidence intervals (CIs) for any CHIP associated with early-life events. Minimally adjusted models included age, sex, ethnicity, smoking and the top 10 genetic principal components (PCs). Covariates that potentially interacted with CHIP mutation, including body mass index (BMI), alcohol consumption, physical activity, coronary heart disease, hypertension and type 2 diabetes mellitus, were adjusted in fully adjusted models. Ideal physical activity was defined as meeting the guideline of at least 150 min of moderate-intensity activity, 75 min of vigorous-intensity activity, or an equivalent combination per week. Further adjusted models included education and the Townsend deprivation index, which might be influenced by early-life events. Second, we studied the associations with gene-specific CHIP subtypes. Subgroup analyses were performed by sex. Finally, several sensitivity analyses were conducted, including those excluding individuals with hematologic malignancies within the first year of follow-up, incorporating diseases as time-varying covariates, and using restricted cubic splines (RCSs) to assess potential nonlinear relationships.

Given the distinct inferential aims, we used a tiered approach to multiple testing. Prespecified, hypothesis-driven associations between early-life factors and CHIP were interpreted using effect estimates and nominal two-sided *p* values, with findings considered suggestive. For the high-dimensional proteomic screening across a large number of proteins, Benjamini–Hochberg correction was applied to control the false discovery rate. Nominal two-sided *p* values < 0.05 were considered statistically significant unless otherwise specified. All statistical analyses were conducted using R software version 4.1.0.

## 3. Results

### 3.1. Participant Characteristics

At baseline, 17,513 (3.8%) participants with any CHIP were identified, among which the most common subtype was *DNMT3A* (10,957, 2.4%), followed by *TET2* (2575, 0.6%), and *ASXL1* (1613, 0.4%), with 736 (0.2%) participants harboring mutations in at least two genes simultaneously. Additionally, 509 (0.1%) participants had spliceosome-gene-specific CHIP, including *PRPF8*, *SF3B1*, *SRSF2*, *U2AF1*, and *ZRSR2.* The baseline characteristics were compared according to the occurrence of CHIP (Table 1). Compared with participants free of CHIP, the CHIP carriers were more likely to be older, current or previous smokers, with higher daily alcohol consumption but lower physical activity and education levels. Moreover, participants with CHIP mutations had higher prevalences of type 2 diabetes, chronic kidney disease, hypertension, or coronary heart disease (all *p* < 0.05).

### 3.2. Associations of Early-Life Factors with Occurrence of Any CHIP Mutation

**Figure 1:**
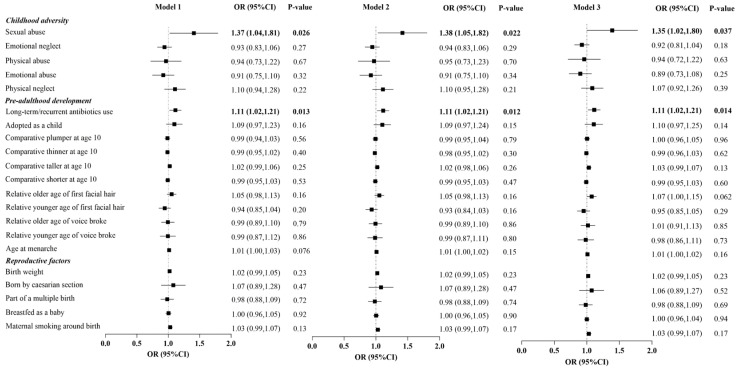
ORs (95% CIs) for CHIP mutations according to early-life events. Model 1 was adjusted for age, sex, ethnicity, smoking status and the top 10 genetic principal components (10 PCs). Model 2 was adjusted for age, sex, ethnicity, smoking status, 10 PCs, body mass index, daily alcohol consumption, ideal physical activity, baseline coronary heart disease, baseline hypertension, and baseline type 2 diabetes. Model 3 was adjusted for age, sex, ethnicity, smoking status, 10 PCs, body mass index, daily alcohol consumption, ideal physical activity, baseline coronary heart disease, baseline hypertension, baseline type 2 diabetes, Townsend deprivation index, and education level. Abbreviations: OR, odds ratio; CI, confidence interval; CHIP, clonal hematopoiesis of indeterminate potential.

Figure 1 illustrates the associations between early-life factors and any CHIP mutation. Among five childhood adversities, sexual abuse was associated with a 35% increased odds of CHIP mutations (OR 1.35, 95% CI 1.02–1.80), in a fully adjusted model including social factors, health behaviors and diseases in adulthood. Additionally, long-term/recurrent antibiotic use as a child or teenager was positively associated with CHIP mutations (OR 1.11, 95% CI 1.02–1.21) among seven pre-adulthood development factors. However, no significant association was observed between five reproductive factors and CHIP mutations.

When analyzing large CHIP (VAF ≥ 10%) and small CHIP (VAF < 10%), the associations of sexual abuse (OR 1.41, 95% CI 1.00–1.99) and long-term/recurrent antibiotic use (OR 1.13, 95% CI 1.02–1.25) with CHIP mutations were strengthened in large CHIP. Moreover, higher birth weight (OR 1.04, 95% CI 1.00–1.09) was specifically associated with large CHIP mutations, while maternal smoking (OR 1.08, 95% CI 1.01–1.15) and being adopted as a child (OR 1.27, 95% CI 1.03–1.56) were only associated with small CHIP (Table S3).

### 3.3. Associations of Early-Life Factors with Gene-Specific CHIP

**Figure 2:**
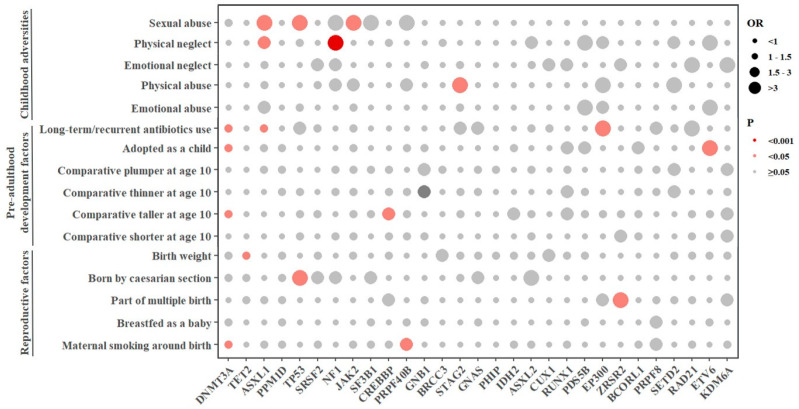
Heterogenous associations of early-life events with gene-specific CHIP mutations. All models were adjusted for age, sex, ethnicity, smoking status, the top 10 genetic principal components (10 PCs), body mass index, daily alcohol consumption, ideal physical activity, baseline coronary heart disease, baseline hypertension, baseline type 2 diabetes, Townsend deprivation index, and education level. Abbreviations: CHIP, clonal hematopoiesis of indeterminate potential.

We further revealed the heterogeneous associations of early-life events with gene-specific CHIP mutations (Figure 2 and Table S4). Sexual abuse, a CHIP-related childhood adversity, was strongly associated with *ASXL1*-CHIP (OR 3.02, 95% CI 1.41–6.46), followed by *JAK2* (OR 7.62, 95% CI 1.79–32.46) and *TP53* (OR 4.44, 95% CI 1.06–18.56). Although the remaining four childhood adversities were not associated with overall CHIP mutations, they were observed to influence gene-specific CHIP mutations. For example, physical abuse was positively associated with *STAG2*-CHIP occurrence, while physical neglect was positively associated with CHIP mutations in *ASXL1* and *NF1* (Table S4).

The significant association of long-term/recurrent antibiotic use with CHIP mutations was primarily driven by three gene-specific CHIP mutations, including *DNMT3A* (OR 1.11, 95% CI 1.01–1.23), *ASXL1* (OR 1.35, 95% CI 1.01–1.81) and *EP300* (OR 3.50, 95% CI 1.03–11.87). Additionally, compared with individuals with about average body size at age 10 years old, thinner individuals were observed to be associated with *GNB1*-CHIP mutations, while taller individuals were associated with CHIP mutations in *CREBBP* and *DNMT3A*. Being adopted as a child was linked to CHIP mutations in *DNMT3A* and *ETV6* (Table S4).

Additionally, maternal smoking was significantly associated with *DNMT3A* and *PRPF40B* CHIP mutations. Increased birth weight, being part of a multiple birth, and being born by caesarian section were associated with CHIP mutations in *TET2*, *ZRSR2*, and *TP53*, respectively (Table S4).

Notably, the significant associations of early-life events with gene-specific CHIP mutations exhibited distinct patterns between high and low frequency (Table S5). For *DNMT3A*, the gene-specific CHIP most frequently associated with early-life events, significant associations of small *DNMT3A*-CHIP were consistent with those for overall *DNMT3A*-CHIP. In contrast, for *NF1*-CHIP, significant associations with early-life events were predominantly found in large *NF1*-CHIP.

### 3.4. Sex-Stratified Analysis of Association Between Early-Life Events and CHIP Mutations

When stratified by sex, sexual abuse and long-term/recurrent antibiotic use were potentially associated with CHIP mutations in males, rather than females, while no interaction was observed between sex and early-life events (Table S6).

For gene-specific CHIP mutations, the association between sexual abuse and *ASXL1*-CHIP mutations was significant in males, whereas the associations with mutations in *JAK2* and *TP53* were significant in females. The associations of long-term/recurrent antibiotic use with CHIP mutations in *DNMT3A*, *ASXL1* and *EP300* were attenuated after stratifying by gender, with potential associations with *DNMT3A* and *EP300* in males and *ASXL1* in females. More details of sex-specific associations between early-life events and gene-specific CHIP mutations are displayed in Table S7. Additionally, the questionnaires related to sexual development were separated by gender. Premature sexual development in males, indicated by first facial hair or voice break, was associated with CHIP mutations in *EP300*, *GNAS* and *BCORL1*, while delayed development was linked to CHIP mutations in *NF1*, *BRCC3*, *PRPF8*, *KDM6A*, and *RUNX1*. In females, premature sexual development, indicated by menarche, was associated with *CREBBP*-CHIP mutations, while delayed development was associated with *TET2*-CHIP mutations.

### 3.5. Circulating Proteomic Biomarkers Shared Between Early-Life Factors and CHIP Mutations

We identified circulating proteomic biomarkers associated with CHIP mutations, in which the association patterns differed among gene-specific CHIP. Furthermore, some biomarkers were associated with early-life factors (Table S8). Concretely, sexual abuse was associated with higher B2M levels (β = 0.12), and higher B2M was associated with higher odds of *JAK2*-CHIP (OR = 2.23). For birth weight and *TET2*-CHIP, 28 proteins showed consistent directions of association. Most proteins were inversely associated with birth weight and were also associated with lower odds of *TET2*-CHIP, including HGF (β = −0.040; OR = 0.58), LCN2 (β = −0.021; OR = 0.44), VWF (β = −0.033; OR = 0.71), and CCL5 (β = −0.045; OR = 0.81). In contrast, FLT3, SELL, and CD1C were positively associated with birth weight and higher odds of *TET2*-CHIP, with ORs of 2.25, 3.67, and 13.46, respectively. Maternal smoking around birth was associated with higher ITIH4 levels (β = 0.011), and higher ITIH4 was associated with higher odds of *PRPF40B*-CHIP (OR = 6.05). All associations described above had *p* values < 0.001. Overall, we identified six pairs of early-life factors and gene-specific CHIP mutations that exhibited significant shared biomarkers after Benjamini–Hochberg correction.

### 3.6. Sensitivity Analysis

The robustness of the associations was verified by sensitivity analyses (Table S9). Consistent associations were observed when excluding individuals having hematologic malignancies within the first year of follow-up. Further, we observed that the associations were slightly attenuated when diseases were modeled as time-varying covariates. In the primary analysis, both birth weight and menarche were treated as continuous variables when examining their associations with CHIP mutations. In the sensitivity analysis, we employed RCS to explore nonlinear relationships, but no significant nonlinear associations were found (Figure S2). However, the relationship between menarche and *PRPF40B*-CHIP appeared to follow a reverse L-shaped curve, with an increase in *PRPF40B*-CHIP prevalence for those with menarche occurring after the age of 16, suggesting a potential nonlinear relationship.

## 4. Discussion

In this large cohort study, we identified associations between several early-life factors and WES-detectable CHIP in adulthood. The associations were not uniform across CHIP driver genes and appeared to differ by sex in some stratified analyses. Sexual abuse and long-term/recurrent antibiotic use showed the most consistent associations with CHIP, particularly gene-specific CHIP subtypes. Proteomic analyses further identified circulating proteins that were associated with both early-life exposures and specific CHIP subtypes, including B2M for sexual abuse and *JAK2*-CHIP, and a broader set of biomarkers shared by birth weight and *TET2*-CHIP. These findings should be interpreted as hypothesis-generating rather than causal, but they suggest that early-life exposures may be relevant to later-life clonal hematopoiesis through immune, inflammatory, infectious, developmental, or hematopoietic pathways.

Among childhood adversities, sexual abuse showed the most consistent association with CHIP. This finding is biologically plausible because sexual abuse has been linked to persistent immune dysregulation and low-grade inflammation, including higher levels of C-reactive protein, interleukin-6, and tumor necrosis factor-α [23]. Inflammatory milieus, interleukin-1 signaling, and chronic stress can promote hematopoietic stem and progenitor cell activation, clonal selection or expansion [24,25,26]. The proteomic finding implicated B2M in the association between sexual abuse and *JAK2*-CHIP. B2M alterations have been implicated in immune evasion during PD-1 therapy [27], suggesting that the immune system may play an important role in these associations. However, this interpretation remains exploratory. Childhood adversity is associated with lower educational attainment, smoking, obesity, and other adverse health behaviors in later life [28,29], which are themselves related to CHIP [30]. Although the association persisted after adjustment for these covariates, residual confounding and unmeasured pathways cannot be excluded.

Long-term/recurrent antibiotic use was also associated with any CHIP and displayed gene- and sex-specific patterns. Recurrent antibiotic treatment can impair murine hematopoiesis by depleting the intestinal microbiota, reducing bone marrow regulatory T cells, and altering microbiota-derived metabolites and type I interferon signaling pathways that support normal hematopoiesis [31,32,33]. However, an alternative explanation is confounding by indication. Long-term antibiotic use may serve as a surrogate marker for frequent infections or immune dysfunction. This possibility is supported by recent evidence that infection frequency is associated with incident CHIP in the general population, as well as studies linking immune dysregulation, such as human immunodeficiency virus infection, to a higher prevalence of CHIP [16,34]. Therefore, this association should be interpreted as hypothesis-generating, and future studies with detailed infection history, antibiotic indications, immune phenotyping, and longitudinal CHIP assessment are needed.

Maternal smoking around birth and being adopted as a child were associated with *DNMT3A*-CHIP. Pubertal development factors were associated with *CREBBP-*, *EP300-*, *NF1-*, or *TET2*-related CHIP subtypes. These findings may reflect early developmental programming, sex-hormone exposure, psychosocial stress, or DNA damage-related pathways, but they should be interpreted cautiously because the biological mechanisms are indirect and the number of gene-specific events may be limited. Although CHIP mutations generally accumulate with age, clonal expansion occurs more rapidly in youth and slows in older age [35], raising the possibility that early-life exposures could influence the later detectability of specific mutant clones. Nevertheless, the gene-specific and sex-stratified findings in this study should primarily be viewed as exploratory signals requiring replication.

Birth weight showed a distinct proteomic signature in relation to *TET2*-CHIP. We identified 28 circulating biomarkers shared by birth weight and *TET2*-CHIP that showed consistent directions of associations, predominantly enriched in pathways related to cell death and immune processes [36]. Prior studies have shown that higher birth weight is associated with an increased risk of developing CHIP in midlife [37]. Telomere biology may provide a plausible mechanism, as higher birth weight has been linked to longer telomere length [38], and longer telomeres may permit greater clonal expansion of *DNMT3A-*, *TET2-*, and *JAK2*-mutant hematopoietic clones before critical telomere attrition occurs [39,40]. These findings provide valuable insight into the associations of early-life events with the development of CHIP, warranting further investigation.

Several limitations should be considered. First, participants in the U.K. Biobank cohort are more likely to be representative of a “healthy volunteer” population, which could lead to an underestimation of the strength of the observed associations in our study. Second, early-life exposures were retrospectively self-reported several decades after the events occurred. Sensitive experiences such as childhood abuse may be underreported because of stigma, avoidance, or reluctance to disclose trauma. If such underreporting was largely nondifferential with respect to CHIP status, the resulting exposure misclassification would likely attenuate the observed associations. However, recall and disclosure may also be influenced by mental health status. Mental health may partly lie on the pathway from childhood adversity to later inflammatory, behavioral, and biological dysregulation, but it may also affect reporting. Therefore, differential reporting cannot be fully excluded. Third, objective records of childhood abuse, childhood infections, or antibiotic indications were not available, limiting our ability to validate self-reported exposures or separate antibiotic exposure from infection burden. Fourth, about 90% of the study participants were of European ancestry, limiting the generalizability of the findings to other ethnic groups. Fifth, because CHIP was identified from WES rather than from targeted deep sequencing, low-VAF clones may have been missed, and the true CHIP burden may be underestimated. Finally, gene-specific and sex-stratified analyses may involve sparse events; these findings should be interpreted as exploratory.

## 5. Conclusions

Early-life factors, especially sexual abuse and long-term/recurrent antibiotic use, were independently associated with CHIP mutations. Based on gene-specific analyses, these findings were especially pronounced in CHIP mutations in *DNMT3A, ASXL1* and *JAK2*. Additionally, the identification of shared circulating biomarkers provided insights into potential mechanisms underlying the associations between early-life factors and CHIP. Given the associations of CHIP mutations with aging and adverse health outcomes, these findings may help inform future studies on CHIP susceptibility, particularly among individuals with adverse early-life factors.

## Figures and Tables

**Table 1:** Baseline characteristics of the study participants according to CHIP status.

Characteristic	Overall	Non-CHIP	Any CHIP	*p*-Value
Number of participants, No. (%)	456,658	439,145 (96.2)	17,513 (3.8)	
Age, mean (SD), y	56.5 (8.1)	56.4 (8.1)	60.6 (6.8)	<0.001
Sex, No. (%)				
Female	247,612 (54.2)	238,167 (54.2)	9445 (53.9)	0.430
Male	209,046 (45.8)	200,978 (45.8)	8068 (46.1)	
Race, No. (%)				
Non-White	61,829 (13.6)	59,460 (13.5)	2369 (13.5)	0.961
White	394,829 (86.5)	379,685 (86.5)	15,144 (86.5)	
Smoking status, No. (%)				
Never	408,448 (89.4)	393,124 (89.5)	15,324 (87.5)	<0.001
Previous	35,243 (7.7)	33,574 (7.6)	1669 (9.5)	
Current	12,507 (2.7)	12,006 (2.7)	501 (2.9)	
Unknown	460 (0.1)	441 (0.1)	19 (0.1)	
Body mass index, mean (SD), kg/m^2^	27.4 (4.6)	27.4 (4.6)	27.4 (4.5)	0.298
Daily alcohol consumption, mean (SD), g/week	16.5 (19.3)	16.5 (19.3)	17.0 (20.1)	<0.001
Ideal physical activity, No. (%)				
No	164,031 (35.9)	157,511 (35.9)	6520 (37.2)	<0.001
Yes	292,627 (64.1)	281,634 (64.1)	10,993 (62.8)	
CHD, No. (%)				
No	434,494 (95.1)	418,110 (95.2)	16,384 (93.6)	<0.001
Yes	22,164 (4.9)	21,035 (4.8)	1129 (6.4)	
Hypertension, No. (%)				
No	208,703 (45.7)	201,906 (46.0)	6797 (38.8)	<0.001
Yes	247,955 (54.3)	237,239 (54.0)	10,716 (61.2)	
T2D, No. (%)				
No	432,704 (94.8)	416,288 (94.8)	16,416 (93.7)	<0.001
Yes	23,954 (5.2)	22,857 (5.2)	1097 (6.3)	
Education, No. (%)				
Non-college	222,731 (48.8)	213,667 (48.7)	9064 (51.8)	<0.001
College	233,927 (51.2)	225,478 (51.3)	8449 (48.2)	
Townsend deprivation index, mean (SD)	−1.4 (2.9)	−1.4 (2.9)	−1.4 (2.9)	0.741

Data are presented as mean (SD) or n (%). The *p*-values were obtained by comparisons of baseline characteristics between any CHIP and non-CHIP using the Student’s t-test for continuous variables or Pearson chi-squared test for categorical variables.

## Data Availability

The data underlying this article are available from the U.K. Biobank, at https://biobank.ndph.ox.ac.uk/ukb/index.cgi (accessed on 9 January 2025). The datasets were derived from sources in the public domain: https://ukbiobank.dnanexus.com/ (accessed on 9 January 2025). The practical method to ascertain CHIP is available at putative somatic variant identification: https://github.com/briansha/Cloud_Development/tree/master/DNANexus/Mutect2 (accessed on 9 January 2025); U2AF1 putative variant identification: https://github.com/weinstockj/pileup_region (accessed on 9 January 2025); annotation and filtering pipeline: https://github.com/briansha/Annovar_Whitelist_Filter_WDL (accessed on 9 January 2025). The script for restricted cubic splines is available at https://github.com/harrelfe/rms (accessed on 9 January 2025).

## Supplementary Materials

The following supporting information can be downloaded at https://www.mdpi.com/article/10.3390/biomedicines14061366/s1.

## Abbreviations

CHIP, clonal hematopoiesis of indeterminate potential; T2D, type 2 diabetes; CHD, coronary heart disease.
