# Supplementary material for: Adverse Early-Life Factors Associated with Clonal Hematopoiesis of Indeterminate Potential in Later Life
Source: Biomedicines. 2026 Jun 17;14(6):1366. doi: 10.3390/biomedicines14061366 (PMC13297359; doi:10.3390/biomedicines14061366)
Supplement: Supplementary file 1 [file biomedicines-14-01366-s001.zip › biomedicines-4252329-supplementary.pdf]

Adverse early-life factors associated with clonal hematopoiesis of indeterminate potential in later life

Content

Figure S1 The flowchart of study design

Table S1 17 early-life events form UK Biobank included in this study

Table S2 74 driven genes for clonal hematopoiesis

Table S3 Associations of early-life events with occurrence of small CHIP (VAF <10%) and large CHIP (VAF ≥10%)

Table S4 Associations of early-life events with occurrence of gene-specific CHIP

Table S5 Associations of early-life events with occurrence of gene-specific small CHIP and large CHIP

Table S6 Sex-specific associations between early-life events and occurrence of CHIP

Table S7 Sex-specific associations between early-life events and occurrence of gene-specific CHIP

Table S8 Circulating proteomic biomarkers shared between early-life factors and CHIP mutations

Table S9 Sensitivity analyses of associations between early-life events and incident CHIP

Figure S2 Nonlinear relationships of birth weight and age at menarche with incident CHIP in RCS

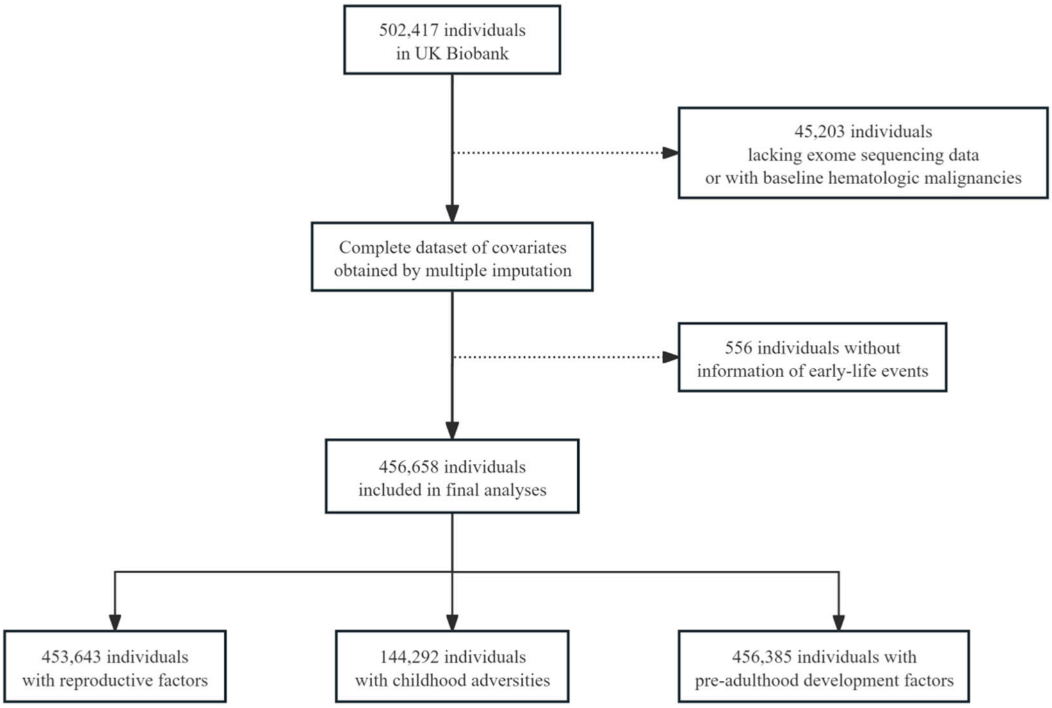

Figure S1 The flowchart of study design

The solid line represents the analysis process and the dotted line represents the exclusion process.

Table S1 17 early-life events form UK Biobank included in this study

Early-life events	Field ID and Description	Definition
Childhood adversities		
Emotional neglect	20489 Felt loved as a child	Sometimes/rarely/never felt loved
Sexual abuse	20490 Sexually molested as a child	Often/very often sexually molested
Physical abuse	20488 Physically abused by family as a child	Often/very often physically abused
Emotional abuse	20487 Felt hated by family member as a child	Often/very often felt hated
Physical neglect	20491 Someone to take to doctor when needed as a child	Sometimes/rarely/never taken to doctor when needed
Pre-adulthood development factors		
Adopted as a child	1767 Adopted as a child	Participant answered “YES”
Comparative plumper at age 10	1687 Comparative body size at age 10	Participant answered “Plumper”
Comparative thinner at age 10	1687 Comparative body size at age 10	Participant answered “Thinner”
Comparative taller at age 10	1697 Comparative height size at age 10	Participant answered “Taller”
Comparative shorter at age 10	1697 Comparative height size at age 10	Participant answered “Shorter”
Relative younger age of first facial hair	2375 Relative age of first facial hair	Participant answered “Younger than average”
Relative older age of first facial hair	2375 Relative age of first facial hair	Participant answered “Older than average”
Relative younger age of voice broke	2385 Relative age voice broke	Participant answered “Younger than average”
Relative older age of voice broke	2385 Relative age voice broke	Participant answered “Older than average”
Age at menarche	2714 Age when periods started (menarche)	Continuous variable
Long-term/recurrent antibiotics use	21067 Long-term/recurrent antibiotics as child or teenager	Participant answered “YES”
Reproductive factors		
Birth weight	20022 Birth weight	Continuous variable
Born by caesarian section	21066 Born by caesarian section	Participant answered “YES”
Part of a multiple birth	1777 Part of a multiple birth	Participant answered “YES”
Breastfed as a baby	1677 Breastfed as a baby	Participant answered “No”
Maternal smoking around birth	1787 Maternal smoking around birth	Participant answered “YES”

Table S2 74 driven genes for clonal hematopoiesis

Gene	Mutations	NCBI RefSeq
ASXL1	Frameshift/nonsense/splice-site in exon 11-12	NM_015338
ASXL2	Frameshift/nonsense/splice-site in exon 11-12	NM_018263
BCOR	Frameshift/nonsense/splice-site	NM_001123385
BCORL1	Frameshift/nonsense/splice-site	NM_021946
BRAF	G464E, G464V, G466E, G466V, G469R, G469E, G469A, G469V, V471F, V472S, L485W, N581S, I582M, I592M, I592V, D594N, D594G, D594V, D594E, F595L, F595S, G596R, L597V, L597S, L597Q, L597R, A598V, V600M, V600L, V600K, V600R, V600E, V600A, V600G, V600D, K601E, K601N, R603*, W604R, W604G, S605G, S605F, S605N, G606E, G606A, G606V, H608R, H608L, G615R, S616P, S616F, L618S, L618W	NM_004333
BRCC3	Frameshift/nonsense/splice-site	NM_024332
CBL	RING finger missense p.381-421	NM_005188
CBLB	RING finger missense p.372-412	NM_170662
CEBPA	Frameshift/nonsense/splice-site	NM_004364
CREBBP	Frameshift/nonsense/splice-site, D1435E, R1446L, R1446H, R1446C, Y1450C, P1476R, Y1482H, H1487Y, W1502C, Y1503D, Y1503H, Y1503F, S1680del	NM_004380
CSF1R	L301F, L301S, Y969C, Y969N, Y969F, Y969H, Y969D	NM_005211
CSF3R	T615A, T618I, truncating c.741-791	NM_000760
CTCF	Frameshift/nonsense, R377C, R377H, P378A, P378L	NM_006565
CUX1	Frameshift/nonsense	NM_181552
DNMT3A	Frameshift/nonsense/splice-site, F290I, F290C, V296M, P307S, P307R, R326H, R326L, R326C, R326S, G332R, G332E, V339A, V339M, V339G, L344Q, L344P, R366P, R366H, R366G, A368T, A368V, R379H, R379C, I407T, I407N, I407S, F414L, F414S, F414C, A462V, K468R, C497G, C497Y, Q527H, Q527P, Y533C, S535F, C537G, C537R, G543A, G543S, G543C, L547H, L547P, L547F, M548I, M548K, G550R, W581R, W581G, W581C, R604Q, R604W, R635W, R635Q, S638F, G646V, G646E, L653W, L653F, I655N, V657A, V657M, R659H, Y660C, V665G, V665L, M674V, R676W, R676Q, G685R, G685E, G685A, D686Y, D686G, R688H, G699R, G699S, G699D, P700L, P700S, P700R, P700Q, P700T, P700A, D702N, D702Y, V704M, V704G, I705F, I705T, I705S, I705N, G707D, G707V, C710S, C710Y, S714C, V716D, V716F, V716I, N717S, N717I, P718L, R720H, R720G, K721R, K721T, Y724C, R729Q, R729W, R729G, F731C, F731L, F731Y, F731I, F732del, F732C, F732S, F732L, E733G, E733A, F734L, F734C, Y735C, Y735N, Y735S, R736H, R736C, R736P, L737H, L737V, L737F, L737R, A741V, P742P, P743R, P743L, R749C, R749L, R749H, R749G, F751L, F751C, F752del, F752C, F752L, F752I, F752V, W753G, W753C, W753R, L754P, L754R, L754H, F755S, F755I, F755L, M761I, M761V, G762C, V763I, S770L, S770W, S770P, R771Q, F772I, F772V, L773R, L773V, E774K, E774D, E774G, I780T, D781G, R792H, W795C, W795L, G796D, G796V, N797Y, N797H, N797S, P799S, P799R, P799H, R803S, R803W, P804L, P804S, K826R, S828N, K829R, T835M, N838D, K841Q, Q842E, P849L, D857N, W860R, E863D, F868S, G869S, G869V, M880V, S881R, S881I, R882H, R882P, R882C, R882G, A884P, A884V, Q886R, L889P, L889R, G890D, G890R, G890S, V895M, P896L, V897G, V897D, R899L, R899H, R899C, L901R, L901H, P904L, F909C, P904Q, A910P, C911R, C911Y	NM_022552
EED	Frameshift/nonsense/splice-site, L240Q, I363M	NM_003797
EP300	Frameshift/nonsense/splice_site, VF1148_1149del, D1399N, D1399Y, P1452L, Y1467N, Y1467H, Y1467C, R1627W, A1629V	NM_001429
ETNK1	N155(244)S, N155(244)T, N155(244)K	NM_018638
ETV6	Frameshift/nonsense/splice-site	NM_001987
EZH2	Frameshift/nonsense/splice-site, Q62R, N102S, F145S, F145C, F145Y, F145L, G159R, E164D, R202Q, K238E, E244K, R283Q, H292R, P488S, R497Q, R561H, T568I, K629E, Y641N, Y641H, Y641S, Y641C, Y641F, D659Y, D659G, V674M, A677G, A677V, R679C, R679H, R685C, R685H, A687V, N688I, N688K, H689Y, S690P, I708V, I708T, I708M, E720K, E740K	NM_001203247
FLT3	V579A, V592A, V592I, F594L, FY590-591GD, D835Y, D835H, D835E, del835	NM_004119
GATA1	Frameshift/nonsense/splice-site	NM_002049
GATA2	Frameshift/nonsense/splice-site, R293Q, N317H, A318T, A318V, A318G, G320D, L321P, L321F, L321V, Q328P, R330Q, R361L, L359V, A372T, R384G, R384K	NM_001145661
GATA3	Frameshift/nonsense/splice-site ZNF domain, R276W, R276Q, N286T, L348V,	NM_001002295
GNA13	I34T, G57S, S62F, M68K, Q134R, Y145F, L152F, E167D, Q169H, R264H, E273K, V322G, V362G, L371F	NM_006572
GNAS	R201S, R201C, R201H, R201L, Q227K, Q227R, Q227L, Q227H, R374C	NM_000516
GNB1	K57N, K57M, K57E, K57T, I80T, I80N	NM_002074
IDH1	R132C, R132G, R132H, R132L, R132P, R132V, V178I	NM_005896
IDH2	R140W, R140Q, R140L, R140G, R172W, R172G, R172K, R172T, R172M, R172N, R172S	NM_002168
IKZF1	Frameshift/nonsense	NM_006060
IKZF2	Frameshift/nonsense	NM_016260
IKZF3	Frameshift/nonsense	NM_012481
JAK1	T478A, T478S, V623A, A634D, L653F, R724H, R724Q, R724P, T782M, L783F	NM_002227
JAK2	N533D, N533Y, N533S, H538R, K539E, K539L, I540T, I540V, V617F, R683S,	NM_004972

	R683G, del/ins537-539L, del/ins538-539L, del/ins540-543MK, del/ins540-544MK, del/ins541-543K, del542-543, del543-544, ins11546-547	
JAK3	M511T, M511I, A572V, A572T, A573V, R657Q, V715I, V715A	NM_000215
KDM6A	Frameshift/nonsense/splice-site, del419	NM_021140
KIT	ins503, V559A, V559D, V559G, V559I, V560D, V560A, V560G, V560E, del560, E561K, del579, P627L, P627T, R634W, K642E, K642Q, V654A, V654E, H697Y, H697D, E761D, K807R, D816H, D816Y, D816F, D816I, D816V, D816H, del551-559	NM_000222
KRAS	G12D, G12A, G12E, G12V, G13D, G13C, G13Y, G13F, G13R, G13A, G13V, G13E, V14I, T58I, G60D, G60A, G60V, Q61K, Q61E, Q61P, Q61R, Q61L, Q61H, K117E, K117N, A146T, A146P, A146V	NM_033360
LUC7L2	Frameshift/nonsense/splice-site	NM_016019
KMT2A	Frameshift/nonsense	NM_005933
KMT2D	Frameshift/nonsense	NM_003482
MPL	S505G, S505N, S505C, L510P, del513, W515A, W515R, W515K, W515S, W515L, A519T, A519V, Y591D, W515-518KT	NM_005373
NF1	Frameshift/nonsense	NM_000267
NPM1	Frameshift p.W288fs (insertion at c.859_860, 860_861, 862_863, 863_864)	NM_002520
NRAS	G12S, G12R, G12C, G12N, G12P, G12Y, G12D, G12A, G12V, G12E, G13S, G13R, G13C, G13N, G13P, G13Y, G13D, G13A, G13V, G13E, G60E, G60R, Q61R, Q61L, Q61K, Q61P, Q61H, Q61Q	NM_002524
PDS5B	Frameshift/nonsense/splice-site, R1292Q	NM_015032
PDSS2	Frameshift/nonsense	NM_020381
PHF6	Frameshift/nonsense/splice-site, A40D, M125I, S246Y, F263L, R274Q, C297Y, H302Y, H329L	NM_001015877
PHIP	Frameshift/nonsense/splice-site	NM_017934
PPM1D	Frameshift/nonsense, exon 5 or 6	NM_003620
PRPF40B	Frameshift/nonsense/splice-site, P15H, M58I, P405L, P562S,	NM_001031698
PRPF8	M1307I, C1594W, D1598Y, D1598N, D1598V	NM_006445
PTEN	Frameshift/nonsense/splice-site, D24G, R47G, F56V, L57W, H61R, K66N, Y68H, C71Y, F81C, Y88C, D92G, D92V, D92E, H93Y, H93D, H93Q, N94I, P95L, I101T, C105F, C105S, D107Y, L112V, H123Y, C124R, C124S, K125E, A126D, K128N, R130G, R130Q, R130L, G132D, I135V, I135K, C136R, C136F, K144Q, A151T, D153Y, D153N, Y155H, Y155C, R159K, R159S, R161K, R161I, G165R, G165E, S170N, S170I, R173C, Y174D, Y177C, H196Y, R234W, G251C, D252Y, F271S, D326G	NM_000314
PTPN11	G60V, G60R, G60A, D61Y, D61V, D61G, Y63C, E69K, E69G, E69D, E69Q, F71L, F71K, A72T, A72V, A72D, T73I, E76K, E76Q, E76M, E76A, E76G, E139G, E139D, N308D, N308T, N339S, P491L, S502P, S502A, S502L, G503V, G503G, G503A, G503E, Q506P, T507A, T507K	NM_002834
RAD21	Frameshift/nonsense/splice-site, R65Q, H208R, Q474R	NM_006265
RUNX1	Frameshift/nonsense/splice-site, S73F, H78Q, H78L, R80C, R80P, R80H, L85Q, P86L, P86H, S114L, D133Y, L134P, R135G, R135K, R135S, R139Q, R142S, A165V, R174Q, R177L, R177Q, A224T, D171G, D171V, D171N, R205W, R223C	NM_001001890
SETBP1	D868N, D868T, S869N, G870S, I871T, D880N, D880Q	NM_015559
SETD2	Frameshift/nonsense, V1190M	NM_014159
SETDB1	Frameshift/nonsense, K715E	NM_001145415
SF1	Frameshift/nonsense/splice-site, T454M, Y476C, A508G	NM_004630
SF3A1	Frameshift/nonsense/splice-site, A57S, M117I, K166T, Y271C	NM_005877
SF3B1	G347V, R387W, R387Q, E592K, E622D, Y623C, R625L, R625C, R625G, H662Q, H662D, T663I, K666N, K666T, K666E, K666R, K700E, V701F, A708T, G740R, G740E, A744P, D781G, E783K, R831Q, L833F, E862K, R957Q	NM_012433
SRSF2	Y44H, P95H, P95L, P95T, P95R, P95A, P107H, P95fs	NM_003016
SMC1A	K190T, R586W, M689V, R807H, R1090H, R1090C	NM_006306
SMC3	Frameshift/nonsense, R155I, Q367E, D392V, K571R, R661P, G662C	NM_005445
STAG1	Frameshift/nonsense/splice-site, H1085Y	NM_005862
STAG2	Frameshift/nonsense/splice-site	NM_006603
SUZ12	Frameshift/nonsense	NM_015355
TET2	Frameshift/nonsense/splice-site, missense mutations in catalytic domains (p.1104-1481 and 1843-2002)	NM_001127208
TP53	Frameshift/nonsense/splice-site, S46F, G105C, G105R, G105D, G108S, G108C, R110L, R110C, T118A, T118R, T118I, S127F, S127Y, L130V, L130F, K132Q, K132E, K132W, K132R, K132M, K132N, F134V. F134L, F134S, C135W, C135S, C135F, C135G, C135Y, Q136K, Q136E, Q136P, Q136R, Q136L, Q136H, A138P, A138V, A138A, A138T, T140I, C141R, C141G, C141A, C141Y, C141S, C141F, C141W, V143M, V143A, V143E, L145Q, W146C, W146L, L145R, V147G, P151T, P151A, P151S, P151H, P151R, P152S, P152R, P152L, T155P, T155A, V157F, R158H, R158L, A159V, A159P, A159S, A159D, A161T, A161D, Y163N, Y163H, Y163D, Y163S, Y163C, K164E, K164M, K164N, K164P, H168Y, H168P, H168R, H168L, H168Q,	NM_001126112

	M169I, M169T, M169V, E171K, E171Q, E171G, E171A, E171V, E171D, V172D, V173M, V173L, V173G, R174W, R175G, R175C, R175H, C176R, C176G, C176Y, C176F, C176S, P177R, P177R, P177L, H178D, H178P, H178Q, H179Y, H179R, H179Q, R181C, R181Y, D186G, G187S, P190L, P190T, H193N, H193P, H193L, H193R, L194F, L194R, I195F, I195N, I195T, R196P, V197L, G199V, Y205N, Y205C, Y205H, D208V, R213Q, R213P, R213L, R213Q, H214D, H214R, S215G, S215I, S215R, V216M, V217G, Y220N, Y220H, Y220S, Y220C, E224D, I232F, I232N, I232T, I232S, Y234N, Y234H, Y234S, Y234C, Y236N, Y236H, Y236C, M237V, M237K, M237I, C238R, C238G, C238Y, C238W, N239T, N239S, S241Y, S241C, S241F, C242G, C242Y, C242S, C242F, G244S, G244C, G244D, G245S, G245R, G245C, G245D, G245A, G245V, G245S, M246V, M246K, M246R, M246I, N247I, R248W, R248G, R248Q, R249G, R249W, R249T, R249M, P250L, I251N, L252P, I254S, I255F, I255N, I255S, L257Q, L257P, E258K, E258Q, D259Y, S261T, G262D, G262V, L265P, G266R, G266E, G266V, R267W, R267Q, R267P, E271K, V272M, V272L, R273S, R273G, R273C, R273H, R273P, R273L, V274F, V274D, V274A, V274G, V274L, C275Y, C275S, C275F, A276P, C277F, C277Y, P278T, P278A, P278S, P278H, P278R, P278L, G279E, R280G, R280K, R280T, R280I, R280S, D281N, D281H, D281Y, D281G, D281E, R282G, R282W, R282Q, R282P, E285K, E285V, E286G, E286V, E286K, K320N, L330R, G334V, R337C, R337L, A347T, L348F, T377P	
U2AF1	D14G, S34F, S34Y, R35L, R156H, R156Q, Q157R, Q157P	NM_001025203
U2AF2	R18W, Q143L, M144I, L187V, Q190L	NM_007279
WT1	Frameshift/nonsense/splice-site	NM_024426
ZRSR2	Frameshift/nonsense, R126P, E133G, C181F, H191Y, I202N, F239V, F239Y, N261Y, C280R, C302R, C326R, H330R, N382K	NM_005089

RefSeq, standard transcript number.

Table S3 Associations of early-life events with occurrence of small CHIP (VAF <10%) and large CHIP (VAF ≥10%)

Early-life events	Small CHIP (VAF <10%)		Large CHIP (VAF ≥10%)	
	**OR (95% CI)**	***P*-value**	**OR (95% CI)**	***P*-value**
Childhood adversities				
Emotional neglect	**0.79 (0.63,1.00)**	**0.048**	0.98 (0.84,1.14)	0.79
Sexual abuse	1.25 (0.76,2.05)	0.39	**1.41 (1.00,1.99)**	**0.047**
Physical abuse	0.77 (0.47,1.27)	0.31	1.02 (0.75,1.39)	0.89
Emotional abuse	0.75 (0.52,1.07)	0.11	0.97 (0.77,1.22)	0.76
Physical neglect	1.02 (0.77,1.35)	0.90	1.10 (0.91,1.33)	0.33
Pre-adulthood development factors				
Long-term/recurrent antibiotics use	1.09 (0.94,1.25)	0.26	**1.13 (1.02,1.25)**	**0.024**
Adopted as a child	**1.27 (1.03,1.56)**	**0.027**	1.03 (0.88,1.20)	0.73
Comparative plumper at age 10	0.98 (0.90,1.07)	0.68	1.01 (0.95,1.07)	0.74
Comparative thinner at age 10	0.99 (0.93,1.05)	0.72	0.99 (0.95,1.04)	0.71
Comparative taller at age 10	1.03 (0.97,1.11)	0.32	1.03 (0.98,1.08)	0.25
Comparative shorter at age 10	0.97 (0.90,1.04)	0.38	1.00 (0.95,1.05)	0.97
Relative older age of first facial hair	1.07 (0.94,1.22)	0.33	1.07 (0.98,1.17)	0.11
Relative younger age of first facial hair	1.08 (0.91,1.29)	0.38	0.88 (0.78,1.01)	0.059
Relative older age of voice broke	0.90 (0.73,1.11)	0.32	1.06 (0.93,1.21)	0.38
Relative younger age of voice broke	1.16 (0.94,1.43)	0.17	0.90 (0.76,1.05)	0.18
Age at menarche	1.00 (0.97,1.02)	0.79	1.02 (1.00,1.03)	0.06
Reproductive factors				
Birth weight	0.97 (0.92,1.03)	0.33	**1.04 (1.00,1.09)**	**0.034**
Born by caesarian section	1.05 (0.77,1.42)	0.78	1.07 (0.86,1.33)	0.55
Part of a multiple birth	0.95 (0.78,1.16)	0.62	0.99 (0.87,1.13)	0.86
Breastfed as a baby	0.97 (0.90,1.05)	0.42	1.01 (0.96,1.07)	0.65
Maternal smoking around birth	**1.08 (1.01,1.15)**	**0.025**	1.00 (0.96,1.05)	0.86

All models were adjusted for age, sex, ethnicity, smoking status, top10 genetic principal components (10 PCs), body mass index, daily alcohol consumption, ideal physical activity, baseline coronary heart disease, baseline hypertension, baseline type 2 diabetes, Townsend deprivation index, and education level.

Abbreviations: OR, odds ratio; CI, confidence interval; CHIP, clonal haematopoiesis of indeterminate potential; VAF, variant allele frequency.

Table S4 Associations of early-life events with occurrence of gene-specific CHIP

Early-life events	CHIP mutations	Cases	OR (95% CI)	*P*-value
Childhood adversities				
Sexual abuse	ASXL1	7†	3.02 (1.41,6.46)	0.0043
	JAK2	2†	7.62 (1.79,32.46)	0.006
	TP53	2†	4.44 (1.06,18.56)	0.041
Physical abuse	STAG2	2†	5.33 (1.21,23.49)	0.027
Physical neglect	ASXL1	370	1.79 (1.17,2.75)	0.0075
	NF1	33	6.60 (2.86,15.24)	<0.001
Pre-adulthood development factors				
Long-term/recurrent antibiotics use	DNMT3A	466	1.11 (1.01,1.23)	0.04
	ASXL1	56	1.35 (1.01,1.81)	0.041
	EP300	4†	3.50 (1.03,11.87)	0.044
Adopted as a child	DNMT3A	188	1.18 (1.01,1.38)	0.033
	ETV6	2†	4.76 (1.13,19.97)	0.033
Comparative thinner at age 10	GNB1	53	2.29 (1.49,3.54)	<0.001
Comparative taller at age 10	DNMT3A	2768	1.05 (1.00,1.10)	0.041
	CREBBP	31	1.87 (1.14,3.05)	0.012
Relative older age of first facial hair	NF1	18†	1.96 (1.15,3.34)	0.013
Relative older age of voice broke	BRCC3	5†	3.48 (1.31,9.24)	0.012
	RUNX1	3†	3.57 (1.01,12.56)	0.048
	PRPF8	3†	4.93 (1.39,17.49)	0.014
	KDM6A	2†	5.58 (1.12,27.69)	0.036
Relative younger age of voice broke	GNAS	4†	3.67 (1.28,10.53)	0.016
	EP300	4†	3.86 (1.31,11.38)	0.015
	BCORL1	2†	5.50 (1.20,25.20)	0.028
Age at menarche	TET2	/	1.04 (1.01,1.08)	0.026
	PRPF40B	/	1.29 (1.12,1.49)	<0.001
Reproductive factors				
Birth weight	TET2	/	1.10 (1.01,1.20)	0.023
Born by caesarian section	TP53	5†	3.70 (1.48,9.27)	0.0051
Part of a multiple birth	ZRSR2	3†	3.34 (1.03,10.83)	0.044
Maternal smoking around birth	DNMT3A	2712	1.05 (1.01,1.11)	0.029
	PRPF40B	41	1.69 (1.12,2.54)	0.012

All models were adjusted for age, sex, ethnicity, smoking status, top10 genetic principal components (10 PCs), body mass index, daily alcohol consumption, ideal physical activity, baseline coronary heart disease, baseline hypertension, baseline type 2 diabetes, Townsend deprivation index, and education level. † Exploratory association due to fewer than 20 cases.

Abbreviations: OR, odds ratio; CI, confidence interval; CHIP, clonal hematopoiesis of indeterminate potential.

Table S5 Associations of early-life events with occurrence of gene-specific small CHIP and large CHIP

Early-life events	CHIP mutations	Small CHIP (VAF <10%)			Large CHIP (VAF ≥10%)		
		Cases	OR (95% CI)	*P*-value	Cases	OR (95% CI)	*P*-value
Childhood adversities							
Sexual abuse	ASXL1	3†	**4.05 (1.27,12.98)**	**0.018**	4†	2.54 (0.93,6.88)	0.068
	TP53	1†	**19.91 (2.30,172.68)**	**0.0066**	1†	2.50 (0.34,18.39)	0.37
	JAK2	/	/	/	2†	**7.62 (1.79,32.46)**	**0.006**
Physical abuse	STAG2	/	/	/	2†	**6.01 (1.35,26.77)**	**0.019**
Physical neglect	ASXL1	108	1.57 (0.69,3.61)	0.28	262	**1.87 (1.14,3.08)**	**0.013**
	NF1	/	/	/	29	**7.19 (3.08,16.8)**	**<0.001**
Pre-adulthood development factors							
Long-term/recurrent antibiotics use	DNMT3A	186	1.06 (0.9,1.24)	0.49	280	**1.15 (1.01,1.32)**	**0.034**
	ASXL1	19†	1.39 (0.83,2.31)	0.21	37	1.34 (0.94,1.90)	0.11
	EP300	/	/	/	4†	**4.70 (1.31,16.91)**	**0.018**
Adopted as a child	DNMT3A	80	**1.29 (1.02,1.64)**	**0.032**	108	1.11 (0.91,1.36)	0.29
	ETV6	/	/	/	2†	**5.61 (1.33,23.69)**	**0.019**
Comparative thinner at age 10	GNB1	/	/	/	53	**2.29 (1.49,3.54)**	**<0.001**
Comparative taller at age 10	DNMT3A	1074	1.06 (0.98,1.14)	0.13	1694	1.05 (0.98,1.11)	0.15
	CREBBP	4†	0.93 (0.28,3.03)	0.90	27	**2.19 (1.27,3.79)**	**0.0049**
Relative older age of first facial hair	NF1	1†	2.42 (0.24,23.99)	0.45	17†	**1.94 (1.13,3.36)**	**0.017**
Relative older age of voice broke	BRCC3	/	/	/	5†	**3.56 (1.34,9.50)**	**0.011**
	RUNX1	/	/	/	3†	**4.11 (1.14,14.89)**	**0.031**
	PRPF8	/	/	/	3†	**4.93 (1.39,17.49)**	**0.014**
	KDM6A	/	/	/	2†	**8.58 (1.61,45.60)**	**0.012**
Relative younger age of voice broke	GNAS	/	/	/	4†	1.11 (0.26,4.68)	0.89
	EP300	1†	1.61 (0.19,13.68)	0.66	3†	2.73 (0.77,9.71)	0.12
	BCORL1	/	/	/	2†	4.52 (0.94,21.6)	0.059
Age at menarche	TET2	/	**1.14 (1.04,1.24)**	**0.0033**	/	1.02 (0.98,1.06)	0.27
	PRPF40B	/	0.49 (0.22,1.11)	0.086	/	**1.33 (1.15,1.54)**	**<0.001**
Reproductive factors							
Birth weight	TET2	/	0.94 (0.76,1.15)	0.54	/	**1.14 (1.04,1.25)**	**0.0057**
Born by caesarian section	TP53	/	/	/	5†	**4.58 (1.82,11.55)**	**0.0013**
Part of a multiple birth	ZRSR2	/	/	/	3†	**3.60 (1.11,11.69)**	**0.033**
Maternal smoking around birth	DNMT3A	1070	**1.10 (1.02,1.18)**	**0.017**	1642	1.03 (0.97,1.09)	0.35
	PRPF40B	/	/	/	41	**1.75 (1.16,2.65)**	**0.0075**

All models were adjusted for age, sex, ethnicity, smoking status, top10 genetic principal components (10 PCs), body mass index, daily alcohol consumption, ideal physical activity, baseline coronary heart disease, baseline hypertension, baseline type 2 diabetes, Townsend deprivation index, and education level. Certain gene-specific CHIP groups had few cases, even no case, resulting in OR values ranging from 0 to infinity and P-values of 1. Such results are represented as "/" in the table. † Exploratory association due to fewer than 20 cases.

Abbreviations: OR, odds ratio; CI, confidence interval; CHIP, clonal haematopoiesis of indeterminate potential; VAF, variant allele frequency.

Table S6 Sex-specific associations between early-life events and occurrence of CHIP

Early-life events	Female		Male		$P_{interaction}$
	OR (95% CI)	*P*-value	OR (95% CI)	*P*-value	
Childhood adversities					
Emotional neglect	0.92 (0.78,1.08)	0.30	0.92 (0.75,1.12)	0.39	0.91
Sexual abuse	*1.33 (0.97,1.83)*	*0.075*	1.49 (0.78,2.83)	0.23	0.75
Physical abuse	1.02 (0.74,1.42)	0.89	0.81 (0.52,1.27)	0.37	0.46
Emotional abuse	0.94 (0.75,1.18)	0.60	0.76 (0.51,1.12)	0.17	0.34
Physical neglect	1.05 (0.85,1.29)	0.68	1.11 (0.87,1.41)	0.41	0.54
Pre-adulthood development factors					
Long-term/recurrent antibiotics use	*1.10 (1.00,1.22)*	*0.058*	1.12 (0.97,1.31)	0.12	0.91
Adopted as a child	1.11 (0.94,1.32)	0.22	1.09 (0.9,1.31)	0.39	0.99
Comparative plumper at age 10	1.00 (0.94,1.06)	0.91	1.01 (0.94,1.09)	0.81	0.69
Comparative thinner at age 10	1.01 (0.96,1.06)	0.73	0.98 (0.93,1.03)	0.43	0.29
Comparative taller at age 10	1.05 (0.99,1.10)	0.091	1.01 (0.96,1.07)	0.70	0.25
Comparative shorter at age 10	1.01 (0.95,1.07)	0.76	0.97 (0.91,1.03)	0.27	0.27
Reproductive factors					
Birth weight	1.01 (0.97,1.06)	0.54	1.02 (0.97,1.08)	0.35	0.55
Born by caesarian section	1.09 (0.86,1.38)	0.50	1.04 (0.79,1.37)	0.79	0.73
Part of a multiple birth	1.05 (0.91,1.22)	0.47	0.89 (0.75,1.05)	0.16	0.14
Breastfed as a baby	1.00 (0.94,1.05)	0.92	1.00 (0.93,1.08)	0.92	0.77
Maternal smoking around birth	1.04 (0.99,1.09)	0.15	1.01 (0.96,1.07)	0.64	0.61

All models were adjusted for age, sex, ethnicity, smoking status, top10 genetic principal components (10 PCs), body mass index, daily alcohol consumption, ideal physical activity, baseline coronary heart disease, baseline hypertension, baseline type 2 diabetes, Townsend deprivation index, and education level. Abbreviations: OR, odds ratio; CI, confidence interval.

Table S7 Sex-specific associations between early-life events and occurrence of gene-specific CHIP

Early-life events	CHIP mutations	Female			Male			$P_{interaction}$
		Cases	OR (95% CI)	*P*-value	Cases	OR (95% CI)	*P*-value	
Childhood adversities								
Sexual abuse	**ASXL1**	2†	1.23 (0.30,5.02)	0.77	5†	**6.75 (2.71,16.82)**	**<0.001**	*0.058*
	**JAK2**	2†	**8.82 (1.97,39.58)**	**0.0045**	/	/	/	/
	**TP53**	2†	**6.80 (1.58,29.23)**	**0.01**	/	/	/	/
	SF3B1	1†	**14.24 (1.72,117.65)**	**0.014**	/	/	/	/
Physical abuse	**STAG2**	2†	**7.19 (1.63,31.77)**	**0.0093**	/	/	/	/
Physical neglect	**ASXL1**	132	*1.84 (0.93,3.65)*	*0.082*	238	**1.76 (1.02,3.04)**	**0.043**	0.87
	**NF1**	12†	**12.30 (3.87,39.08)**	**<0.001**	21	**3.83 (1.10,13.31)**	**0.035**	0.32
	PDS5B	7†	**6.15 (1.26,30.04)**	**0.025**	/	/	/	/
	ETV6	/	/	/	2†	**22.24 (1.61,306.74)**	**0.02**	/
Emotional neglect	GNB1	/	/	/	6†	**5.95 (1.14,31.05)**	**0.035**	/
	CUX1	/	/	/	6†	**5.44 (1.07,27.71)**	**0.041**	/
	RAD21	/	/	/	4†	**10.26 (1.79,58.80)**	**0.0089**	/
Emotional abuse	PDS5B	2†	**5.90 (1.2,29.06)**	**0.029**	/	/	/	/
	ASXL1	5†	1.16 (0.47,2.84)	0.75	8†	**2.15 (1.05,4.39)**	**0.036**	0.34
Pre-adulthood development factors								
Long-term/recurrent antibiotics use	**DNMT3A**	335	1.08 (0.95,1.22)	0.22	131	*1.20 (0.99,1.45)*	*0.062*	0.47
	**ASXL1**	31	*1.48 (0.98,2.22)*	*0.062*	25	1.24 (0.82,1.88)	0.32	0.51
	**EP300**	1†	1.66 (0.16,17.02)	0.67	3†	**5.04 (1.18,21.45)**	**0.029**	0.44
	RAD21	4†	**7.19 (1.55,33.28)**	**0.012**	/	/	/	/
Adopted as a child	**DNMT3A**	105	1.15 (0.94,1.41)	0.18	83	*1.23 (0.97,1.55)*	*0.086*	0.64
	**ETV6**	1†	4.93 (0.65,37.59)	0.12	1†	4.51 (0.59,34.28)	0.15	0.91
Comparative thinner at age 10	**GNB1**	35	**2.25 (1.33,3.81)**	**0.0026**	18†	**2.47 (1.15,5.30)**	**0.02**	0.91
Comparative plumper at age 10	ASXL1	114	**1.33 (1.05,1.68)**	**0.016**	146	0.96 (0.79,1.17)	0.69	*0.083*
	PPM1D	47	**1.60 (1.10,2.33)**	**0.014**	31	0.87 (0.58,1.29)	0.48	**0.034**
Comparative shorter at age 10	NF1	27	**1.83 (1.06,3.13)**	**0.029**	20	1.02 (0.59,1.75)	0.95	0.13
Comparative taller at age 10	**DNMT3A**	1640	*1.06 (1.00,1.13)*	*0.059*	1128	1.04 (0.96,1.11)	0.36	0.50
	**CREBBP**	20	**2.14 (1.15,3.99)**	**0.017**	11†	1.49 (0.66,3.32)	0.33	0.53
	RUNX1	9†	**3.02 (1.07,8.55)**	**0.037**	5†	1.24 (0.41,3.71)	0.70	0.21
Relative older age of first facial hair	NF1	/	/	/	18†	**1.96 (1.15,3.34)**	**0.013**	/
Relative older age of voice broke	BRCC3	/	/	/	5†	**3.48 (1.31,9.24)**	**0.012**	/
	RUNX1	/	/	/	3†	**3.57 (1.01,12.56)**	**0.048**	/
	PRPF8	/	/	/	3†	**4.93 (1.39,17.49)**	**0.014**	/
	KDM6A	/	/	/	2†	**5.58 (1.12,27.69)**	**0.036**	/
Relative younger age of voice broke	GNAS	/	/	/	4†	**3.67 (1.28,10.53)**	**0.016**	/
	EP300	/	/	/	4†	**3.86 (1.31,11.38)**	**0.015**	/
	BCORL1	/	/	/	2†	**5.50 (1.20,25.20)**	**0.028**	/
Age at menarche	TET2	/	**1.04 (1.01,1.08)**	**0.026**	/	/	/	/
	PRPF40B	/	**1.29 (1.12,1.49)**	**<0.001**	/	/	/	/
Reproductive factors								

Birth weight	**TET2**	/	1.07 (0.95,1.20)	0.25	/	**1.14 (1.01,1.29)**	**0.035**	0.39
	BRCC3	/	**1.67 (1.03,2.72)**	**0.039**	/	1.18 (0.51,2.72)	0.69	0.47
	PDS5B	/	**3.17 (1.58,6.39)**	**0.0012**	/	0.60 (0.30,1.20)	0.15	**<0.001**
	PPM1D	/	0.84 (0.63,1.13)	0.24	/	**1.39 (1.08,1.80)**	**0.011**	**0.0077**
	TP53	/	1.25 (0.88,1.76)	0.21	/	**0.63 (0.45,0.88)**	**0.0073**	**0.0059**
Born by caesarian section	**TP53**	2†	2.66 (0.63,11.15)	0.18	3†	**4.90 (1.47,16.33)**	**0.0096**	0.55
Part of a multiple birth	**ZRSR2**	2†	**4.56 (1.06,19.68)**	**0.042**	1†	2.08 (0.28,15.48)	0.48	0.60
	TET2	38	**1.45 (1.04,2.02)**	**0.027**	27	0.90 (0.59,1.38)	0.63	0.10
	CREBBP	4†	**3.42 (1.22,9.62)**	**0.02**	1†	1.19 (0.16,8.82)	0.86	0.86
Maternal smoking around birth	**DNMT3A**	1532	1.04 (0.97,1.10)	0.26	1180	**1.08 (1.00,1.16)**	**0.04**	0.53
	**PRPF40B**	25	**1.88 (1.10,3.22)**	**0.021**	16†	1.47 (0.78,2.77)	0.23	0.36
	ASXL1	163	**1.35 (1.11,1.65)**	**0.0032**	248	0.90 (0.77,1.05)	0.17	**0.0015**
	CREBBP	12†	**2.14 (1.02,4.49)**	**0.043**	3†	0.46 (0.13,1.57)	0.21	0.10
	SRSF2	12†	0.76 (0.36,1.61)	0.48	61	**1.45 (1.04,2.00)**	**0.027**	0.12

All models were adjusted for age, sex, ethnicity, smoking status, top10 genetic principal components (10 PCs), body mass index, daily alcohol consumption, ideal physical activity, baseline coronary heart disease, baseline hypertension, baseline type 2 diabetes, Townsend deprivation index, and education level. Certain gene-specific CHIP groups had few cases, even no case during follow-up, resulting in OR values ranging from 0 to infinity and P-values of 1. Such results were represented as "/" in the table. Genes in bold represented those significantly associated with early-life events in the overall population. † Exploratory association due to fewer than 20 cases.

Abbreviations: OR, odds ratio; CI, confidence interval; CHIP, clonal haematopoiesis of indeterminate potential.

Table S8 Circulating proteomic biomarkers shared between early-life factors and CHIP mutations

Early-life events	Olink protein	CHIP	β	*P*-value	OR	*P*-value
Childhood adversities						
Sexual abuse	B2M	JAK2	0.120	<0.001	2.23	<0.001
Pre-adulthood development factors						
Comparative taller at age 10	LAMA4	GNAS	-0.012	0.005	0.02	<0.001
Relative older age of first facial hair	COMP	NF1	-0.034	<0.001	0.10	<0.001
Age at menarche	C1QTNF1	TET2	-0.007	<0.001	0.64	<0.001
Reproductive factors						
Birth weight	FCER2	TET2	-0.016	0.002	0.70	0.001
	FLT3		0.011	<0.001	2.25	<0.001
	FLT3LG		-0.020	<0.001	0.09	<0.001
	HGF		-0.040	<0.001	0.58	<0.001
	KIR2DL2		-0.029	0.001	0.67	<0.001
	KIR2DL3		-0.031	<0.001	0.68	<0.001
	LAMA4		-0.013	<0.001	0.47	<0.001
	LCN2		-0.021	<0.001	0.44	<0.001
	NID1		-0.020	<0.001	0.52	<0.001
	OLFM4		-0.054	<0.001	0.85	0.002
	PGLYRP1		-0.015	<0.001	0.64	<0.001
	PRSS27		-0.014	0.001	0.46	<0.001
	RETN		-0.019	<0.001	0.55	<0.001
	SCPEP1		-0.024	<0.001	0.65	0.001
	SELL		0.006	0.007	3.67	<0.001
	TNFRSF10C		-0.015	0.003	0.70	<0.001
	TNFRSF11B		-0.019	<0.001	0.46	<0.001
	TPP1		-0.018	<0.001	0.63	<0.001
	VWF		-0.033	<0.001	0.71	<0.001
	C1QTNF1		-0.021	<0.001	0.64	<0.001
	CCL5		-0.045	<0.001	0.81	<0.001
	CCN4		-0.010	0.012	0.50	<0.001
	CD1C		0.009	<0.001	13.46	<0.001
	CDH15		-0.031	<0.001	0.70	<0.001
	ADGRF5		-0.016	<0.001	0.39	<0.001
	CHRDL1		-0.011	<0.001	0.53	<0.001
	DEFA1_DEFA1B		-0.022	<0.001	0.67	<0.001
	EPPK1		-0.022	0.005	0.74	0.001
Maternal smoking around birth	ITIH4	PRPF40B	0.011	<0.001	6.05	<0.001

All models were adjusted for age, sex, ethnicity, smoking status, top10 genetic principal components (10 PCs), body mass index, daily alcohol consumption, ideal physical activity, baseline coronary heart disease, baseline hypertension, baseline type 2 diabetes, Townsend deprivation index, and education level.

Abbreviations: OR, odds ratio; CHIP, clonal haematopoiesis of indeterminate potential.

Table S9 Sensitivity analyses of associations between early-life events and incident CHIP

Early-life events	CHIP as time-varying covariates		Excluding hematologic malignancies within the first year	
	OR (95% CI)	*P*-value	OR (95% CI)	*P*-value
Childhood adversities				
Emotional neglect	0.92 (0.81,1.04)	0.18	0.92 (0.81,1.04)	0.17
Sexual abuse	**1.35 (1.01,1.79)**	**0.041**	**1.36 (1.02,1.80)**	**0.035**
Physical abuse	0.93 (0.72,1.22)	0.61	0.94 (0.72,1.22)	0.65
Emotional abuse	0.89 (0.73,1.08)	0.24	0.89 (0.73,1.09)	0.25
Physical neglect	1.07 (0.92,1.26)	0.38	1.08 (0.92,1.26)	0.37
Pre-adulthood development factors				
Adopted as a child	1.10 (0.97,1.25)	0.13	1.10 (0.97,1.24)	0.16
Comparative plumper at age 10	0.99 (0.94,1.04)	0.62	1.00 (0.96,1.05)	0.93
Comparative thinner at age 10	0.99 (0.96,1.03)	0.75	0.99 (0.96,1.03)	0.64
Comparative taller at age 10	1.03 (0.99,1.07)	0.15	1.03 (0.99,1.07)	0.14
Comparative shorter at age 10	0.99 (0.95,1.03)	0.66	0.99 (0.95,1.03)	0.67
Relative older age of first facial hair	1.07 (0.99,1.15)	0.068	1.07 (0.99,1.15)	0.068
Relative younger age of first facial hair	0.95 (0.85,1.05)	0.29	0.94 (0.85,1.05)	0.27
Relative older age of voice broke	1.01 (0.90,1.13)	0.86	1.01 (0.91,1.13)	0.83
Relative younger age of voice broke	0.98 (0.86,1.11)	0.72	0.98 (0.86,1.11)	0.70
Age at menarche	1.01 (1.00,1.03)	0.058	1.01 (1.00,1.02)	0.14
Long-term/recurrent antibiotics use	**1.11 (1.02,1.21)**	**0.013**	**1.11 (1.02,1.21)**	**0.015**
Reproductive factors				
Birth weight	1.02 (0.99,1.05)	0.26	1.02 (0.99,1.05)	0.27
Born by caesarian section	1.06 (0.89,1.28)	0.50	1.06 (0.89,1.27)	0.51
Part of a multiple birth	0.98 (0.88,1.09)	0.70	0.98 (0.88,1.09)	0.72
Breastfed as a baby	1.00 (0.95,1.04)	0.86	1.00 (0.96,1.04)	0.99
Maternal smoking around birth	1.02 (0.98,1.06)	0.27	1.03 (0.99,1.07)	0.17

All models were adjusted for age, sex, ethnicity, smoking status, top10 genetic principal components (10 PCs), body mass index, daily alcohol consumption, ideal physical activity, baseline coronary heart disease, baseline hypertension, baseline type 2 diabetes, Townsend deprivation index, and education level.

Abbreviations: OR, odds ratio; CI, confidence interval; CHIP, clonal haematopoiesis of indeterminate potential.

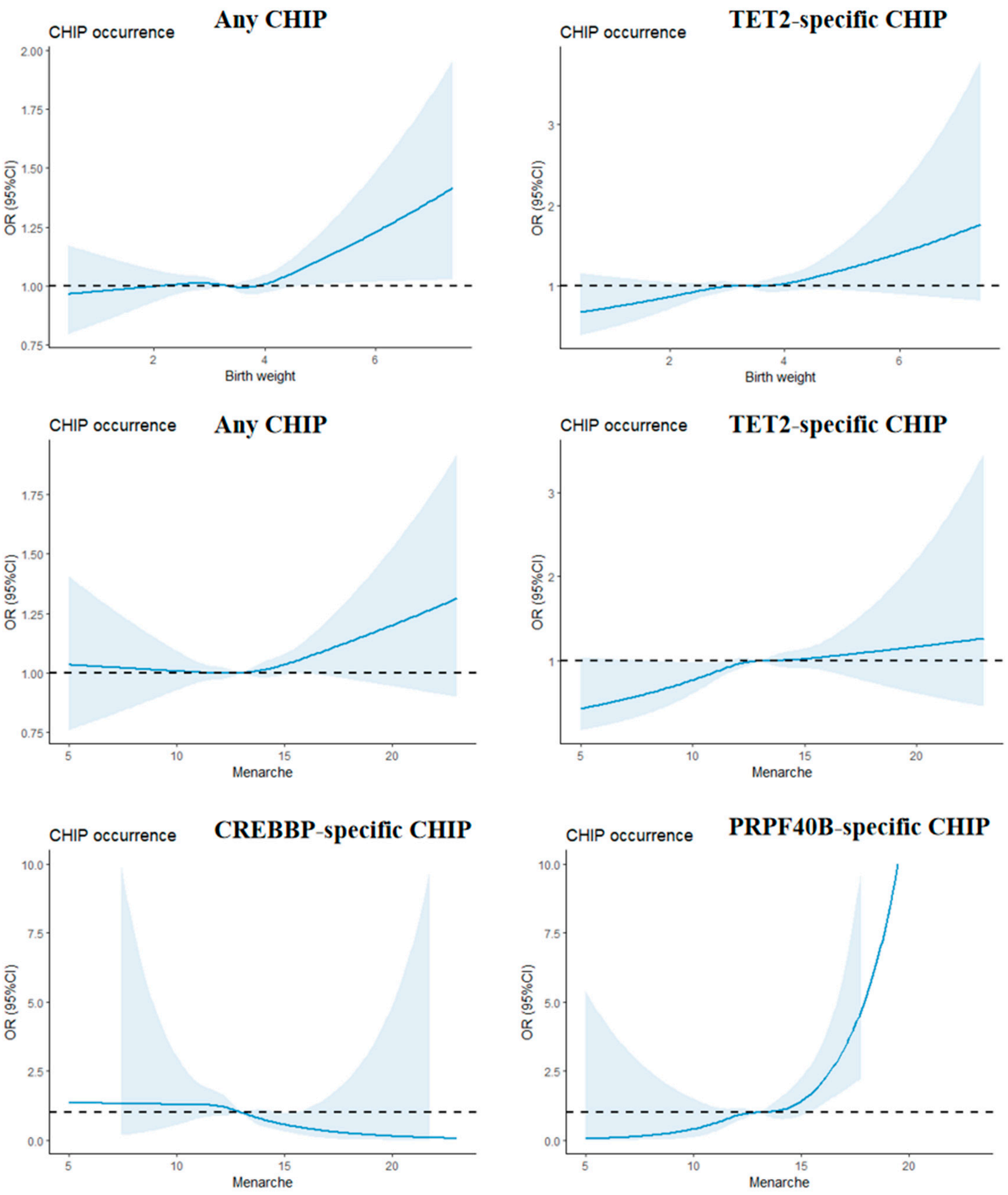

Figure S2 Nonlinear relationships of birth weight and age at menarche with incident CHIP in RCS

Abbreviations: CHIP, clonal haematopoiesis of indeterminate potential.
